# The Halotolerant Probiotic Bacterium *Enterococcus lactis* ASF-2 from Al-Asfar Lake, Saudi Arabia, Reduces Inflammation in Carrageenan-Induced Paw Edema

**DOI:** 10.3390/microorganisms11102415

**Published:** 2023-09-27

**Authors:** Najla Alsaud, Amjad Almajed, Allujayn Lwusaybie, Aljawharah Alsubaie, Hela Alobaidan, Jihad Alessa, Abeer Almousa, Hairul Islam M. Ibrahim, Ashraf Khalifa

**Affiliations:** 1 AlNukhba Modern Schools, Al-Ahsa 31982, Saudi Arabia; 2 Al-Mansourah School, Al-Ahsa 31982, Saudi Arabia; 3 Al-Anjal National Schools, Al-Ahsa 31982, Saudi Arabia; 4 Biological Science Department, College of Science, King Faisal University, P.O. Box 400, Al-Ahsa 31982, Saudi Arabia; 5 Molecular Biology Division, Pondicherry Centre for Biological Sciences and Educational Trust, Pondicherry 605004, India; 6 Botany and Microbiology Department, Faculty of Science, Beni-Suef University, Beni-Suef 62511, Egypt

**Keywords:** *Enterococcus lactis*, Al-Asfar Lake, halotolerant bacteria, inflammatory disorders

## Abstract

Inflammation-related diseases are major causes of mortality and disability worldwide. This study aimed to identify and investigate probiotic bacteria that could be present in Al-Asfar Lake in Al-Ahsa City, Saudi Arabia to prevent the inflammatory responses of carrageenan-induced paw edema. In total, seven active strains were isolated, and three isolates (ASF-1, ASF-2, and ASF-3) exhibited a positive Gram stain and viable growth at 20% NaCl salinity; they also lacked catalase and hemolytic activities and had high levels of cell surface hydrophobicity (CSH). They also demonstrated potent antibacterial activity against *Salmonella typhi* and *Staphylococcus aureus.* These results revealed that ASF-2 had probiotic qualities, and it was selected for further research. ASF-2 demonstrated significant anti-inflammatory effects in an experimental model of carrageenan-induced paw edema; the experimental model showed decreased levels of pro-inflammatory markers, such as interleukin 6 (IL-6), interleukin 17 (IL-17), and transforming growth factor-β (TGF-β), and an increased level of an anti-inflammatory marker (interferon gamma (IFN-γ)). Animals in the control group saw a 45% decrease in edema when compared to mice in the carrageenan group. When comparing tissue damage and infiltration in the ASF-2-treated and non-treated mice, the histological examination of the sub-planar tissues of the hind leg revealed that the inflamed tissues had healed. The 16S rRNA sequencing method was utilized to establish that ASF-2 is, in fact, *Enterococcus lactis* with a 99.2% sequence similarity. These findings shed further light on ASF-2’s potential as a biocompatible anti-inflammatory medication.

## 1. Introduction

One of the leading causes of mortality and disability throughout the globe is inflammation-related disorders [1]. Treating these disorders using drugs has negative side effects, such as indigestion and stomach ulcers—these can cause internal bleeding and anemia [2] (Warman et al., 2022). The stimulation of tissue with traumatic, viral, post-ischemic, toxic, or autoimmune stimuli can result in inflammation, which is a complicated system of interactions between soluble immune factors and associated cells [3]. These manifestations of inflammation include edema (Paw edema), leukocyte infiltration, and granuloma development. Numerous reactions can be induced or made worse by the intricate processes and mediators involved in the inflammatory response [4]. It is a defensive biological response to harmful stimulation, pathogens, or irritants in vascular tissues that tries to eradicate infectious stimulation. The anti-inflammatory drugs (nonsteroidal anti-inflammatory drugs (NSAIDs)) are used to treat a variety of painful conditions, including arthritis, muscular, and ligament pain. Conventional pharmacological treatments are ineffectual in preventing the onset and progression of numerous inflammatory disorders. They also have a wide array of adverse effects on patients. In order to avoid these drawbacks, it is of interest to find an effective, inexpensive, and safe alternative approach to combat inflammatory disorders.

Probiotics are a promising candidate for this task. Probiotics are live microorganisms that, when administered in adequate amounts, confer a health benefit on the host. Probiotics can be found in various ecological niches, including fermented foods, soil, and water [5]. There is a variety of information in the scientific literature regarding the use of probiotic microorganisms in dietary supplements and food products. Important *LactoBacillus* species, such as *L. casei*, possess well-documented antibacterial, antilipidemic, immunomodulatory, anticancer, antidiabetic, and antiarthritic properties [6,7]. Several novel proposed probiotic strains from diverse resources include *Enterococcus faecium* EA9 from marine shrimp [8], *Limosilactobacillus reuteri* AMBF471 from fermentation, *Streptococcus salivarius* from the respiratory tract [9], *LimosilactoBacillus walteri* (isolated from fecal-contaminated soil), and *Lactobacillus fermentum* TIU19 isolated from Haria beer that is an ethnic traditional rice beverage [10]. There is a growing trend to use probiotic-derived components, which are believed to be safer, more stable, and more effective than completely viable probiotics. One of their advantages over whole viable cells is that antibiotic resistance or virulence genes cannot be transferred between bacterial species [11]. However, additional research is required to demonstrate their efficacy as novel biotherapeutic agents. It is of premium importance to search for novel probiotic strains that might have new and improved benefits from previously unexplored ecological niches such as the Al-Asfar region.

Al-Asfar Lake, also known as the Yellow Lake, is a shallow lake in the wetlands located in the Al-Ahsa oasis of Eastern Saudi Arabia. Few studies have explored the diversity of the microflora existing in Al-Asfar Lake. A number of moderately halophilic and halotolerant bacteria have been reported from the lake. These bacteria, which include members of the genera *Halobacillus*, *Staphylococcus*, and *Pseudomonas*, were found to be able to grow in a wide range of salt concentrations from 0 to 15% NaCl and accumulate the compatible solute betaine at high salinities [12]. Limnological studies conducted on Al-Asfar Lake reported 33 planktonic species that belong to the following four algal classes: Bacillariophyceae, Chlorophyceae, Cyanophyceae, and Euglenophyceae [13]. In addition to the bacteria that have been isolated from Al-Asfar Lake, there are likely many other species that have not yet been identified. Further research is needed to fully understand the microbial diversity, particularly the probiotic strains, of this important ecosystem.

Active research is being conducted to find novel probiotic bacteria. The search for novel probiotics employs a variety of techniques, including functional screening [14]. Functional screening is a method that permits scientists to examine the effects of various microorganisms on human cells and tissues. This is useful for identifying bacteria with potential probiotic properties. Numerous studies [15] (Yadav et al., 2020) have evaluated *Lactobacillus’* immunomodulatory properties. Probiotics have potential benefits, including improved gut health; reduced risk of chronic diseases, such as colitis [5] and multiple sclerosis [16,17]; and improved immune functions [18]. With these facts in mind, the current study aimed to investigate, isolate, characterize, and identify probiotic strains from Al-Asfar Lake. Furthermore, the anti-inflammatory efficiency of the active strains was evaluated using a carrageenan-induced acute inflammatory paradigm.

## 2. Materials and Methods

### 2.1. Collection of Water Samples and Isolation of Bacteria

Water samples (1500 mL) were collected in sterilized plastic containers from the surface water of Al-Alasfar Lake, Al-Ahsa, Eaten Province, Saudi Arabia on 22 August 2022. For bacterial isolation, DeMan Rogosa Sharpe (MRS) medium (Himedia, Mumbai, India) was inoculated with 0.1 mL of a serially diluted water sample. Then, the inoculated MRS plates were kept in a 37 °C oven for 48 h. Pure cultures were obtained by streaking individual colonies onto newly made MRS agar plates.

#### 2.1.1. Characterization Based on Morphological and Biochemical Traits

The isolates were initially screened using the culture conditions, the morphology of the colonies, and the observations made using a microscope. The isolates were tested for their ability to produce catalase enzyme by adding drops of H_2_O_2_ to actively growing cells. The evolution of gas bubbles was taken as a positive result. In the ensuing studies, Gram-positive bacilli were chosen to be the focus. MRS medium was used for subsequent bacterial growth in a 37 °C oven for 48 h.

#### 2.1.2. Identification of the Bacterial Strain Using 16S rRNA Gene Analysis

Following the phenol chloroform technique allowed for the successful isolation of genomic DNA [19]. The amplification of the 16S rDNA region of the extracted DNA required a 27F forward primer (5’-AGA GTT TGA TCM TGG CTC AG-3’) and a 1492R reverse primer (5’-TAC GGY TAC CTT GTT ACG ACT T-3’), both of which were purchased from Sigma-Aldrich (St. Louis, MO, USA) in the United States. The PCR amplification was performed in 25 μL reaction mixtures using an Applied Bioscience thermal cycler. The PCR reaction mixture contained 2.5 μL of PCR buffer (10 × PCR amplification enzyme buffer containing 500 mM KCl, 100 mM Tris-HCl (pH 9.0), and 1% Triton X-100), 0.5 μL of PCR master mix in a concentration of 10 mM, 0.5 μL of each primer in a concentration of 10 μM, 20 μL of deionized water, and 0.5 μL of template DNA (corresponding to approximately 50 to 100 ng of DNA). The PCR conditions were as follows: initial denaturation at 94 °C for 4 min, 30 cycles of denaturation at 94 °C for 1 min each, primer annealing at 55 °C for 30 s and primer extensionYes. at 72 °C for 2 min, and a final step of primer extension at 72 °C for 5 min. The amplified 16S rDNA PCR product was sequenced using the ABI 3730xl 96 capillary system that was outfitted with the Big Dye Terminator v3.1 kit at Macrogen in Seoul, Korea. After sequencing the genes, the Geneious R8 software tool R8.0.2 program (Biomatters Ltd., Auckland, New Zealand) was used to build a consensus sequence of 16S rDNA. This was then followed by a BLAST analysis to locate similar sequences in databases that did not include duplicate information. The database was searched for the 10 sequences that had the greatest similarity and maximum identity scores, which led to the selection of a total of ten best-match sequences. The sequences were aligned with the help of Clustal-W, and then a neighbor-joining tree was built utilizing that information.

### 2.2. Investigation of the Characteristics of Probiotics

#### 2.2.1. Testing the Bacterial Isolates for Growth at High Acidity

The early viability of cells may be affected by pH due to the fact that food must remain in the stomach for at least three hours, where it is exposed to an acidic environment. The pH tolerance of the test isolate was evaluated by transferring 500 µL of the bacterial culture grown in MRS broth to separate test tubes, each of which contained 5 mL of a solution made of PBS. The pH levels in the test tubes were set to 3.0 and 7.20 using 1 N HCl and 1 N NaOH, respectively. The test tubes were heated to a temperature of forty degrees Celsius for four hours. After distributing 100 µL of the bacterial suspension onto MRS agar plates at regular intervals of 1 h, the total number of viable colonies was counted and recorded. The result was presented in the form of log10 colony-forming units per milliliter, abbreviated as log_10_ CFU/mL.

#### 2.2.2. Testing for Tolerance to Simulated Gastric Juice

To obtain the cell pellet, a total volume of 100 µL of the bacterial culture that had been cultivated overnight in MRS broth was subjected to centrifugation for 15 min at a force of 2000× *g*. The pellets were suspended in 10 mL of synthetic gastric juice (the test) and then placed in an incubator at a temperature of 35 °C for 4 h. Pepsin at a concentration of 3 g/L, potassium chloride at a concentration of 7 mM, sodium hydroxide at a concentration of 45 mM, and sodium chloride at a concentration of 125 mM were used to make a simulation of gastric juice [20]. As a control, the cell pellets were likewise injected into PBS maintained at a pH of 7.0 and allowed to continue their incubation. Placing 100 µL of each sample on a freshly prepared MRS agar plate allowed for us to assess at regular intervals the number of live cells present in the test as well as the control.

#### 2.2.3. Testing for Tolerance to Bile Salt

The ability of the bacterial isolates to tolerate bile tolerance was performed in accordance with the procedure outlined in [21]. Exactly, 100 µL of the bacterial culture grown in MRS broth was used to inoculate MRS broth with 0.3% ox-bile salts, and the mixture was then heated to 35 °C for 4 h. In addition, the bacterial culture was injected into a bile-free MRS broth in order to perform a control experiment. The spreading of 100 µL of the samples onto newly prepared MRS agar plates was performed on an hourly basis in order to measure the number of viable cells in the bacterial isolate.

#### 2.2.4. The Hydrophobicity of the Cell Surface

According to Krausova et al. [22], the degree to which bacteria are able to adhere to hydrocarbons may be used as an indicator of how well they adhere to the epithelial cells that line the gastrointestinal system [22]. In order to verify this, the bacterial cultures that had been grown in MRS broth overnight were centrifuged at a force of 2000× *g* for 10 min at 4 °C in order to retrieve the bacterial pellets. A total of 3 mL of the bacterial suspension was added to 1 mL of hydrocarbons (n-hexadecane and toluene), and the mixture was stirred using a vortex for a period of 2 min. For the purpose of phase separation, the suspension was allowed to sit undisturbed for an hour, and then, the aqueous phase was carefully extracted from the mixture. The recording of the final absorbance (OD final) required the utilization of the volume that was left over 22. The following formula was used in order to determine the percentage of hydrophobicity on the cell surface based on the initial and final OD:

Percentage Hydrophobicity (%) = OD_initial_ − OD_final_/OD_initial_ × 100.

#### 2.2.5. Test of Tolerance to NaCl

Streaks of the test isolate were made on MRS broth that had been supplemented with several doses of NaCl (0.5, 1, 2, 3, …, 20% (*w*/*v*)). A control plate, consisting of MRS agar that had not been amended with NaCl, was developed. After 24–72 h of incubation at a temperature of 35 °C, the growth patterns of all of the plates were studied.

### 2.3. Safety Analysis

#### 2.3.1. Hemolytic Activity

A hemolytic test was performed on blood agar plates, which were made by adding 5% sheep blood (obtained from a local butcher shop) on brain heart infusion (BHI) agar. The test was carried out in order to determine whether or not the blood agar plates were hemolytic. On the surface of the newly produced blood agar plates, the test isolate was streaked in a specific pattern. After 24–48 h of incubation at 35 °C, the hemolytic zone was recorded. The hemolysis pattern was evaluated qualitatively based on the developed color of the formed zone around the colony.

#### 2.3.2. Preparation of Extracellular Extract (Lysate) of Selected Probiotics

Supernatants of the fermented probiotic broth were acquired with centrifugation at 4000× *g* for 15 min at 20 °C and then were passed through sterile 0.22 μm pore-size filters (Sartorius, Göttingen, Germany) and freeze dried (Sanyo freezer, Tokyo, Japan). The extracts of the extracellular probiotic lysate were prepared by mixing 1 mg freeze-dried samples in 1 mL methanol, and these were kept in a tight container at 4 °C.

#### 2.3.3. Antimicrobial Activity

The well diffusion technique was used in the course of carrying out the antimicrobial activity test. First, MH agar plates were swabbed on the surface with *Salmonella typhi* and *Staphylococcus aureus* bacterial cultures. Then, 6 mm diameter wells were prepared, and cell-free supernatants (lysate) from the isolated probiotic strains were loaded into the wells (100 μL/well). After incubating the plates at 35 °C for 24–48 h, the diameter of the zone of inhibition was measured in millimeters [23].

### 2.4. Evaluation of DPPH-Based Free Radical Scavenging Activity

The procedure described by Chen et al. [24] was used in order to ascertain the capability of neutralizing the free radicals produced with DPPH. The absorbance at 517 nm was measured after adding 100 μL of the sample solution to 2 mL of a 0.1 mmol/L DPPH anhydrous methanol solution. The combination was then incubated for 15 min in the dark. A blank was created by adding the anhydrous methanol to the DPPH solution; the anhydrous methanol solution was utilized to alter the zero position. The following is how we determined the level of scavenging activity:

DPPH scavenging rate (%) = (AC − AS) × 100AC(1)

where AC is the absorbance value of the blank, and AS is the absorbance value of the sample.

### 2.5. Cell Culture, Stimulation of RAW 264.7 Cells with LPS and Bacteria lysate

The Pondicherry Center for Biological Science and Educational Trust gifted the RAW 264.7 cell line. At a temperature of 37 °C and a carbon dioxide concentration of 5%, the RAW cells were incubated with 10% fetal bovine serum (FBS), 100 μg/mL streptomycin, and 100 μg/mL penicillin. The probiotic cell-free lysate from antimicrobial activity was dissolved in the DMEM medium used for the culturing. In a Petri dish of 10 cm in diameter, the RAW cells were cultivated and then subjected to one of the following treatments: control group, LPS treatment group for 30 min, and LPS+ probiotic lysate (12.5 μg/mL, 25 μg/mL, and 50 μg/mL) group. After the cell culture was finished, the cells from the various treatment groups were collected for assessment of cell viability and cytokine estimation.

### 2.6. Evaluation of the Cell Survival Activity

In order to obtain the cell suspension, well-growing RAW cells were digested with trypsin at a concentration of 0.25%, the cell suspension was then mixed with a complete medium consisting of 10% FBS and 1640 medium, and finally, the cells were injected into 96-well plates. After the cells had grown to 80% of their original size, the probiotic lysate was added to create the final concentrations, which were 12.5 μg/mL, 25 μg/mL, and 50 μg/mL, respectively. This was performed while the cells continued to develop. After that, a control group consisting of nothing but a blank page was formed followed by treatment groups consisting of H_2_O_2_ at 20, 40, 60, 80, and 100 μmol/L. Following that, 10 L of MTS solution was poured into each well, and the plates were then put in an incubator containing CO_2_ for a period of 30 min. Within fifteen min, an enzyme-linked immunoassay analyzer was used to determine the absorbance at a wavelength of 492 nm. The rate of cell survival was compared to the control value and represented as a percentage. The RAW cell viability was measured via the cell proliferation, which was expressed as a percentage of OD_490_ relative to the negative control. The experiment was carried out three times, and the results were averaged together.

Cell survival rate (%) = (a − c)/(b − c) × 100 (a = absorbance at each concentration of the probiotic lysate, b = absorbance at untreated wells, and c = absorbance of the blank).

### 2.7. Effect of a Probiotic Combination on the Anti-Inflammatory Response of Mice with Carrageenan-Induced Paw Edema

The overnight-grown probiotic culture was suspended in citrate buffer (10^8^ mL^−1^) and administered orally to carrageenan-induced mice (0.5 mL per 25 g of mice). The anti-inflammatory activity of the probiotic oral suspension was investigated using a mouse model in accordance with the procedure described in [25]. We acquired male BALB/C mice from an animal facility, college of science, King Faisal University, Saudi Arabia (ethical approval KFU-REC-2023-MAY-ETHICS930), and their body weight was measured to be 28 g. The mice were kept in an environment free of any known pathogens with a humidity value of 60.5%. The KFU Ethical Approval Committee gave its stamp of approval to the experimental methods described here. Following an adaption period of one week to the surrounding environment, the mice were divided at random into four groups (n = 5). Following the administration of the drug for an hour, a newly prepared solution of 0.5 mg/25 μL carrageenan (Sigma-Aldrich, St. Louis, MO, USA) was injected into the sub-plantar tissue of the right hind paw of each mouse. The saline solution was injected into the left hind paw, which served as the control for the experiment. After carrageenan-induced paw edema, the size of the paw edema was measured using a Digimatic Caliper at regular intervals of 90 min for 6 h. The difference in thickness between the right paw and the left paw was measured, computed, and reported. Tissue sections were stained with hematoxylin and eosin (H&E) and examined using a microscope for pathological change.

### 2.8. Assessment of Evolutionary Relationships of Taxa

The evolutionary history was inferred using the minimum evolution method [26]. The optimal tree with the sum of branch length = 0.13553341 is shown. The tree is drawn to scale with branch lengths in the same units as those of the evolutionary distances used to infer the phylogenetic tree. The evolutionary distances were computed using the maximum composite likelihood method) [27] and are in the units of the number of base substitutions per site. The ME tree was searched using the Close-Neighbor-Interchange (CNI) algorithm by Nei and Kumar [28] Molecular Evolution and Phylogenetics, (Oxford University Press, New York 2000) at a search level of 0. The Neighbor-joining algorithm was used to generate the initial tree. The analysis involved 12 nucleotide sequences. All positions containing gaps and missing data were eliminated. There were a total of 662 positions in the final dataset. Evolutionary analyses were conducted in MEGA5 [29].

### 2.9. Statistical Analysis

The statistics program known as graph pad (Ver.8.0) was used to carry out the statistical analysis. A one-way analysis of variance was used to compare the findings, and the level of significance for the differences was set at *p* less than 0.05.

## 3. Results

### 3.1. Bacterial Isolates, Morphological and Biochemical Characterization

A microbiota in a lake environment has a rich source of uncharacterized microbes. In this study, water samples from the Al-Asfar Lake in Al Ahsa, Saudi Arabia were collected and enriched with LB medium (agar plates and broth medium) containing a range of salt concentrations up to 20% NaCl (Table 1). Seven strains of halotolerant or moderately halophilic bacteria were isolated and screened for probiotic characteristics. The results revealed that, out of seven isolates, three showed probiotic characteristics and crossed over the screening strategies. The isolates ASF-2, ASF-3, and AF-4 were Gram-positive, catalase-negative, and hemolytic-negative (Table 1). ASF-2 and ASF-3 displayed growth at 20% NaCl as evidenced by the OD_590_ measurement (0.56 ± 0.15, 0.54 ± 0.1, and 0.41 ± 0.06, respectively). The isolates were active against high salt, high acidic pH, and hydrophobic characters.

### 3.2. In Vitro Characteristics of Bacterial Probiotics

**Figure 1:**
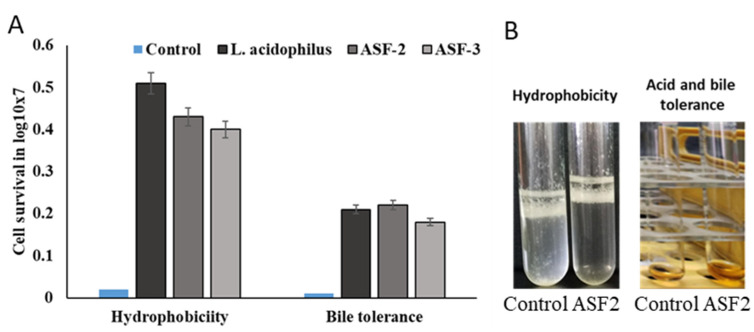
Screening of hydrophobicity and gastric and bile juice tolerance. Evaluation of hydrophobicity for ASF-2 and ASF-3 was carried out using heptane as the nonpolar solvent. (**A**) Cell survival in log10X7 for hydrophobicity and bile tolerance. (**B**) Tubes showing hydrophobicity and acid and bile salt tolerance of ASF-2 where cells tend to aggregate to the upper hydrophobic layer compared to the control. Notice the orange color of the ASF-2 tube indicating well-grown cells compared to the control. Acid and bile tolerance were on all selected isolates using bile sale 0.3% and pH 2.0 of MRS broth.

The pH tolerance test performed on the chosen isolates under various culture conditions showed encouraging results. As the experiment progressed, the isolated *Bacillus* spp. grew steadily at pH values between 1.0 and 3.0 (Figure 1). The bacteria’s development progressively stopped when the pH range fell below 2.0. This discovery demonstrates that isolated probiotic species can endure extremely acidic as well as alkaline environments. All of the strains sustained and were viable at a pH that differed in a highly significant way (*p* < 0.001).

The viability percentage of the cells with and without treatments with bile salts was carried out for a 3 h incubation period in order to ascertain the ability of the bacterial strains (ASF-1, ASF-2, and ASF-3) to survive in the host gastrointestinal tract. All of the tested isolates were able to endure the rising bile salt concentrations (Figure 1). The percentage of viable cells that survived after 3 h of exposure increased dramatically with increasing bile salt concentrations (1.0, 2.5, 5.0, and 7.5%). Both the ASF-1 and ASF-2 exhibited highly significant differences (*p* < 0.001); however, the ASF2 only showed moderately significant variations (*p* < 0.01) between bile concentrations at the survival rates after 3 h.

**Figure 2:**
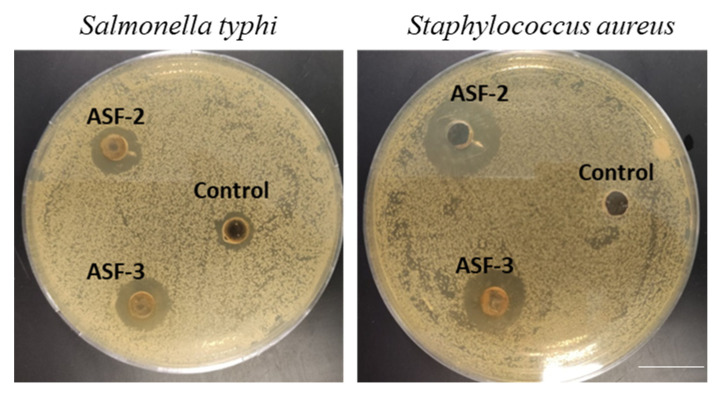
Potential probiotic strains were used to assess the antimicrobial properties using the well diffusion method. The isolates ASF-2 and Asf-3 were used in this study. The cell-free extracts were prepared and tested against *Salmonella typhi* and *Staphyllococcus aureus* (ATCC strains). Scale bar 2 cm.

Potential probiotic strains were assessed for antimicrobial properties against *S. typhi* and *S. aureus* using the well diffusion method. The isolates ASF-2 and ASF-3 were used in this study. ASF-2 showed significant inhibition against the test pathogens (Figure 2).

**Figure 3:**
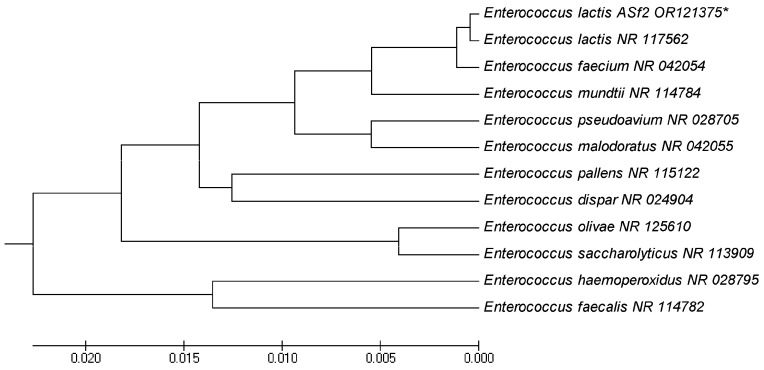
Evolutionary relationships of taxa. The evolutionary history was inferred using the UPGMA. *Enterococcus lactis* OR121375 had 99.2% similarity with *E. lactis* NR117562. Evolutionary analyses were conducted in MEGA5. * Indicates the lactic acid bacteria isolated in this experiment.

Comparative sequence analyses of the 16S rRNA gene of ASF-2 showed that it was closely related to the genus *Enterococcus*, which belongs to Enterococcaceae. *Enterococcus lactis* was the closest species to ASF-2 with 99.2% homology of the 16S rRNA sequence analysis. ASF-2 formed a monophyletic group with the species of the *Enterococcus* group (Figure 3) *E. lactis* (accession No. NR117562).

### 3.3. DPPH-Based Free Radical Scavenging Activity of the Bacterial isolates

**Figure 4:**
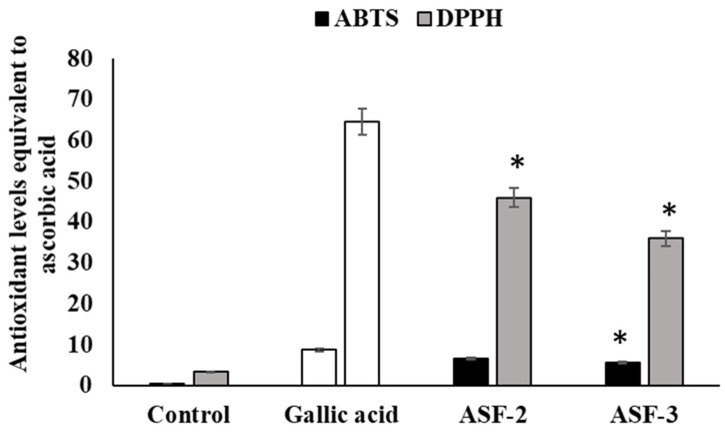
Antioxidant activity of probiotic cell-free extracts. ABTS and DPPH were used to evaluate the antioxidant activity. Gallic acid was used as the positive control of the experiment. Values represent mean ± SEM for triplicate. * Values decreased significantly from control.

By controlling oxidative stress, antioxidant molecules significantly contribute to maintaining the equilibrium of the gut microbiome. Suspensions of the isolated bacterial strains were evaluated for their antioxidant capacities using the DPPH and ABTS free radical scavenging assays (Figure 4). The outcomes showed that all of the strains’ bacterial suspensions had a significant capacity to scavenge DPPH and ABTS free radicals (Figure 4). Among them, ASF-2 showed 45.74% and 6.71%, respectively, which is the highest DPPH and ABTS radical scavenging activity. According to a study, the DPPH radical scavenging capacity of *Lactobacillus plantarum* strains was less than 10%. Additionally, according to a recent study, *L. plantarum* NJAU-01 dramatically reduced lipid peroxidation in mice by boosting enzyme activity. However, isolated *Bacillus* sp. and *Lactobacillus* sp. showed much higher antioxidant activity in this investigation compared to other publications.

### 3.4. Stimulation of RAW 264.7 Cells with LPS and Bacteria Lysate

**Figure 5:**
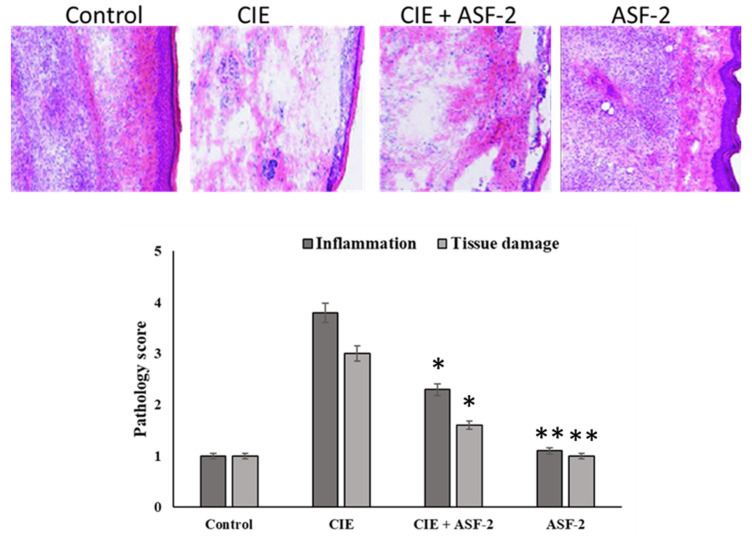
Effect of various drugs on impairment in motility associated with carrageenan-induced inflammation. Edema was induced by injecting 0.1 mL of a 1% solution of carrageenan into the subplantar surface of the right hind paw. The probiotic was administered orally 30 min after injecting the inflammagen. Pathology score and tissue damage were assessed using the H&E staining method. Data are expressed as mean ± standard error of seven mice per group. * Values decreased significantly from control. ** Values decreased in highly significant manner from the control. Group A: control mice; Group B: carrageenan control; Group C: carrageenan with ASF2-fed mice; Group D: ASF2-fed mice.

These isolates were further screened for anti-inflammatory activity in an in vivo mouse model. Edema was induced with carrageenan (0.5 mg/25 μL carrageenan (Sigma-Aldrich, St. Louis, MO, USA)). After 8 days, the paws of the tested mice were extracted and quantified using H&E staining (Figure 5). Carrageenan injection caused a gradual edema in the hind paw that peaked after four hours. The paw thickness of the CIE animals was 3.03 ± 0.04 cm at time zero, and it remained the same after 24 h. At each hour, the paw thickness of the CIE with ASF-2 animals decreased, which was significant at *p* < 0.001. The thickness was 3.03 by 0.040 cm at t = 0 h. The paw thickness of the animals in Group C was 3 ± 0.028 cm, although it slightly increased by the end of the second hour to 3.35 ± 0.0102 cm. The H&E staining revealed the reduced inflammation and tissue damage, and the pale patched eosin staining revealed the exertion of tissue in the sub-planar region of the hind paw (Figure 5).

### 3.5. Effect of a Probiotic Combination on the Anti-Inflammatory Response of Mice with Carrageenan-Induced Paw Edema

**Figure 6:**
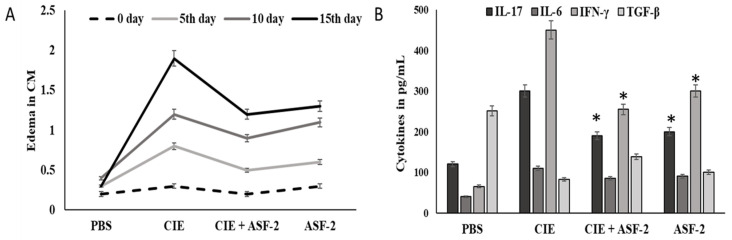
ffect of probiotic bacteria on carrageenan-induced paw edema. (**A**): Size of paw edema (**B**): responses of pro-inflammatory markers, such as interleukin 6 (IL-6), interleukin 17 (IL-17), and transforming growth factor-β (TGF-β), and an increased level of an anti-inflammatory marker (interferon gamma (IFN-γ). PBS: control mice with buffer solution; CIE: carrageenan-induced edema; CIE+ASF-2: carrageenan with ASF2-fed mice; ASF-2: ASF2-fed mice. The results are presented as the mean ± SD (n = 5). * Values decreased significantly from control. Effect of probiotic bacteria on carrageenan-induced paw edema. (**A**,**B**) The results are presented as the mean ± SD (n = 5). * Values decreased significantly from control.

The carrageenan group animals had the highest serum levels of IL17, IL-6, IFN-γ, and TGF-β, which were 280.3, 105, and 437 pg/mL, respectively (Figure 6), while the TGF-β levels in this group were the lowest (74 pg/mL). Treatment with ASF-2 showed a significant reduction in the levels of IL17, IL-6, and IFN-γ in Groups C and D at *p* < 0.01. The lowest level of IL-6 was found in Group D where it was 44.51 ± 0.198 pg/mL, whereas the highest level of TGF-β was found in Group C where it was 420.77 ± 0.27 pg/mL. TNF-, IL-6, and IL-10 levels in the Group E animals were 64.60 ± 0.13 pg/mL and 132.52 ± 0.228 pg/mL. While the TGF-β and IL-6-concentrations were significant, an insignificant change was observed in the IL-6 concentration for the Group C animals.

## 4. Discussion

The current study aimed to isolate, characterize, and identify bacteria with potential probiotic activities from a previously unexplored area, Al-Asfar Lake, Al-Ahsa, and Eastern Province, Saudi Arabia. The results revealed that, out of seven isolates, three showed probiotic characteristics and crossed over the screening strategies. The isolates ASF-1, ASF-2, and AF-4 were Gram-positive, catalase-negative, and hemolytic-negative. They also showed potential hydrophobic characteristics. The three isolates displayed halophilic/halotolerant capability in the presence of 20% NaCl. Bacteria can manage to survive in elevated levels of salinity via production of osmoprotectants, which are molecules that help to protect cells from the effects of high salt concentration. Trehalose helps to maintain the cell’s osmotic balance and prevent it from shrinking or bursting [14]. Probiotics can change the composition of their cell membranes to make them more resistant to the effects of high salt concentration. When exposed to high salt concentration, probiotics can reduce their metabolic activity. This helps them to conserve energy and resources, and it also makes them less susceptible to damage.

In order to be considered a probiotic, a microbe must possess unique multiple features, including hydrophobicity of the cell surface for adhesion, tolerance to bile salts and acids, antibiotic susceptibility, and antimicrobial activity. The catalase test for ASF-1 was negative, meaning that this strain does not produce the catalase enzyme, which is necessary to convert hydrogen peroxide into water and oxygen. This finding suggests that the strain can cope with the conditions of low-oxygen environments. These results are similar to those reported by Saroha et al. [30], who reported on Gram-positive, catalase-negative *Limosilactobacillus walteri* [30].

It is important to screen bacteria for hemolytic activity to initially ensure their safety. ASF-1 had a negative hemolytic response, indicating that the isolate did not cause the lysis of red blood cells. This is a desirable trait in probiotics, as hemolytic bacteria can damage cells in the gut and lead to inflammation. This finding is consistent with previous reports of *Lactobacillus fermentum*, *Enterococcus faecium*, and *Enterococcus hire* isolated from human salivary and fecal sources [31]. It is worth mentioning that there are two main types of hemolysis: alpha hemolysis and beta hemolysis. Alpha hemolysis causes the red blood cells to swell and crenate with greenish zones around the colonies. Beta hemolysis causes the complete lysis of red blood cells, resulting in a clear zone around the bacteria on the blood agar plate. ASF-1 is a non-hemolytic isolate.

The stomach is a very acidic environment with a pH of 1.5–3.5. This acidity can kill many types of bacteria, including probiotics. Therefore, it is important for probiotics to be acid tolerant in order to survive the journey through the stomach and reach the small intestine, where they can colonize and provide their benefits.

Probiotic strains must be able to survive stomach acid and bile salts in order to reach their target in the intestine. Low-pH conditions and bile salts can damage or kill probiotic bacteria. In our study, ASF-1 was able to survive bile salts, acidic pH, and artificial gastric juice. These results are similar to those of a previous study that found that the probiotic *Bacillus subtilis* was also able to survive these conditions [32]. Another common feature of probiotic strains is cell surface hydrophobicity (CSF). CSF is a property of probiotic bacteria that is associated with their ability to adhere to intestinal cells and colonize the gut. ASF-1 displayed a considerable level of CSF. One possible explanation is that probiotic bacteria with a high degree of hydrophobicity have cell surfaces that have fatty acids and other hydrophobic molecules, which help the bacteria to attach to the fatty acids that are present on the surface of intestinal cells. A similar observation was obtained in [33].

ASF-2 exhibited antibiosis against *Salmonella typhi* and *Staphylococcus aureus*, indicating its biocidal impact by inhibiting the pathogen’s metabolism and reproduction via impairing vital processes, such as protein synthesis, cell signaling DNA replication, and membrane permeability [34]. Similar observations were reported with *Lactobacillus* spp. [35,36]. *Salmonella* is a type of bacteria that can cause food poisoning, while *Staphylococcus aureus* is a type of bacteria that can cause skin infections and other health problems. These findings open the door for exploitation of probiotics as promising antibacterial agents for combating pathogenic bacteria. ASF-2 exhibited substantial anti-inflammatory activities as evidenced by the reduction in anti-inflammatory markers (IL17, IL-6, IFN-γ, and TGF-β). One possible explanation of this finding is that ASF-2 could release certain bioactive compounds that block the production of anti-inflammatory enzymes, reduce inflammation by scavenging free radicals, or reducing the production of inflammatory cytokines.

Inflammation is a complex biological response of the host cells to certain stimuli, including irritants, damaged cells, and pathogenic agents. It has protective roles against infection and harmful substances. Carrageenan-induced paw edema is an experimental model for evaluating the anti-inflammatory impacts of natural products. The mechanism by which carrageenan causes inflammation is not fully understood. However, it is believed that carrageenan can bind to the cells lining the gut and trigger the release of inflammatory mediators. Neutrophils are a type of white blood cell that play a significant role in the inflammatory response and repair of damaged tissue via releasing a variety of inflammatory mediators, including cytokines. The results reported herein suggest that anti-inflammatory cytokines are an important part of how ASF-2 works to stop acute inflammatory reactions. However, more research is needed to figure out the exact processes and pathways involved in this process. It has been recently reported that *Lactobacillus* sp. act as an anti-inflammatory agent by modifying the production of the anti-inflammatory cytokines IL-10 and TGF-β [37].

## 5. Conclusions

Herein, we report a probiotic bacterial strain, ASF-2, which was identified as *Enterococcus lactis* at 99.2% sequence similarity. The strains showed multiple probiotic traits, including hydrophobicity of the cell surface for adhesion, tolerance to bile salts and acids, antibiotic susceptibility, and antimicrobial activity. The catalase test and hemolytic activity for ASF-1 were negative. Additionally, the strain displayed substantial anti-inflammatory effects against carrageenan-induced paw edema as an experimental model, as revealed by the suppression of the pro-inflammatory markers and upregulation of the anti-inflammatory markers. However, more research is needed to figure out the exact pathways involved in the anti-inflammatory action. These observations provide primary evidence for the exploitation of ASF-2 as a biocompatible anti-inflammatory agent and human health booster in the future.

## Figures and Tables

**Table 1:** Results of Gram staining, catalase test, growth at low pH and 20% NaCl, and hemolytic activity of the bacterial isolates.

Isolates	Gram Staining	Catalase	pH-3 Growth	Hemolytic Activity	28 °C OD_590_ (NaCl 20%)
ASF-1	+	+	+	+	0.19 ± 0.05
ASF-2	+	−	++	−	0.56 ± 0.15
ASF-3	+	−	++	−	0.54 ± 0.13
ASF-4	−	−	−	−	0.41 ± 0.06
ASF-5	−	−	−	+	0.19 ± 0.07
ASF-6	+	−	+	+	0.26 ± 0.07
ASF-7	+	+	−	++	0.21 ± 0.06

The table values represent the growth in presence and absence. + indicates growth and activity; ++ indicates significant growth and positive activity; and − indicates the absence of growth and negative activity.

## Data Availability

Available from the corresponding author upon request.
